# Triplet supertree heuristics for the tree of life

**DOI:** 10.1186/1471-2105-10-S1-S8

**Published:** 2009-01-30

**Authors:** Harris T Lin, J Gordon Burleigh, Oliver Eulenstein

**Affiliations:** 1 Department of Computer Science, Iowa State University, Ames, IA, USA; 2 National Evolutionary Synthesis Center, Durham, NC, USA; University of Florida, Gainesville, FL, USA

## Abstract

**Background:**

There is much interest in developing fast and accurate supertree methods to infer the tree of life. Supertree methods combine smaller input trees with overlapping sets of taxa to make a comprehensive phylogenetic tree that contains all of the taxa in the input trees. The intrinsically hard triplet supertree problem takes a collection of input species trees and seeks a species tree (supertree) that maximizes the number of triplet subtrees that it shares with the input trees. However, the utility of this supertree problem has been limited by a lack of efficient and effective heuristics.

**Results:**

We introduce fast hill-climbing heuristics for the triplet supertree problem that perform a step-wise search of the tree space, where each step is guided by an exact solution to an instance of a local search problem. To realize time efficient heuristics we designed the first nontrivial algorithms for two standard search problems, which greatly improve on the time complexity to the best known (naïve) solutions by a factor of *n *and *n*^2 ^(the number of taxa in the supertree). These algorithms enable large-scale supertree analyses based on the triplet supertree problem that were previously not possible. We implemented hill-climbing heuristics that are based on our new algorithms, and in analyses of two published supertree data sets, we demonstrate that our new heuristics outperform other standard supertree methods in maximizing the number of triplets shared with the input trees.

**Conclusion:**

With our new heuristics, the triplet supertree problem is now computationally more tractable for large-scale supertree analyses, and it provides a potentially more accurate alternative to existing supertree methods.

## Background

Assembling the tree of life, or the phylogeny of all species, is one of the grand challenges in evolutionary biology. Supertree methods take a collection of species trees with overlapping, but not identical, sets of taxa and return a "supertree" that contains all taxa found in the input trees (e.g., [1-4]). Thus, supertrees provide a way to synthesize small trees into a comprehensive phylogeny representing large sections of the tree of life. Recent supertree analyses have produced the first complete family-level phylogeny of flowering plants [5], and the first phylogeny of nearly all extant mammals [6]. Since the main objective of most supertree analyses is to build extremely large phylogenetic trees by solving intrinsically hard computational problems, the design of efficient and effective heuristics is a critically important part of developing any useful supertree method.

Ideal supertree methods must combine speed and accuracy. By far the most commonly used supertree method is matrix representation with parsimony (MRP; [7,8]). MRP converts a collection of input trees into a binary character matrix, and then performs a parsimony analysis on a matrix representation of the input trees. Thus, MRP analyses can use efficient parsimony heuristics implemented in programs such as PAUP* [9] and TNT [10], making large-scale MRP supertree analyses computationally more tractable. However, the accuracy and performance of MRP are frequently criticized. For example, there is evidence of input tree size and shape biases [11,12], the results can vary depending on the method of matrix representation [11], and the accuracy of the MRP supertrees are not necessarily correlated with the parsimony score [13]. Therefore, there is a need to develop alternate methods that share the advantages of MRP but produce more accurate supertrees.

Since we rarely know the evolutionary history of a group of organisms with certainty, it is usually impossible to assess the accuracy of a supertree based on its similarity to the true species phylogeny. A more practical way to define the accuracy of a supertree is based on the overall similarity of the supertree to the collection of input trees. There are numerous ways to measure the similarity between input trees and the supertree. The intrinsically hard [14] triplet supertree problem measures this similarity based on the common shared triplets, or rooted, binary, 3-taxon trees that are the irreducible unit of phylogenetic information in rooted trees [14]. Specifically, the triplet supertree problem seeks a supertree that shares the most triplets with the input trees.

We introduce hill-climbing heuristics for the triplet supertree problem that make it feasible for truly large-scale phylogenetic analyses. Hill-climbing heuristics have been effectively applied to other intrinsically difficult supertree problems [7,13,15]. They search the space of all possible supertrees guided by a series of exact solutions to instances of a local search problem. The local search problem is to find an optimal phylogenetic tree that shares the most number of triplets with the input trees in the neighborhood of a given tree. The neighborhood is the set of all phylogenetic trees into which the given tree can be transformed by applying a tree edit operation. A variety of different tree edit operations have been proposed [16,17], and two of them, rooted Subtree Pruning and Regrafting (SPR) and Tree Bisection and Reconnection (TBR), have shown much promise for phylogenetic studies [18,19]. However, algorithms for local search problems based on SPR and TBR operations, especially on rooted trees, are still in their infancy. To conduct large-scale phylogenetic analyses, there is much need for effective SPR and TBR based local search problems that can be solved efficiently.

In this work we improve upon the best known (naïve) solutions for the SPR and TBR local search problems by a factor of *n *and *n*^2 ^(the number of taxa in the supertree) respectively. This is especially desirable since standard local search heuristics for the triplet supertree problem typically involve solving several thousand instances of the local search problem. We demonstrate the performance of our new triplet heuristics in a comparative analysis with other standard supertree methods.

### Related work

#### Triplet supertree problem

**Figure 1:**
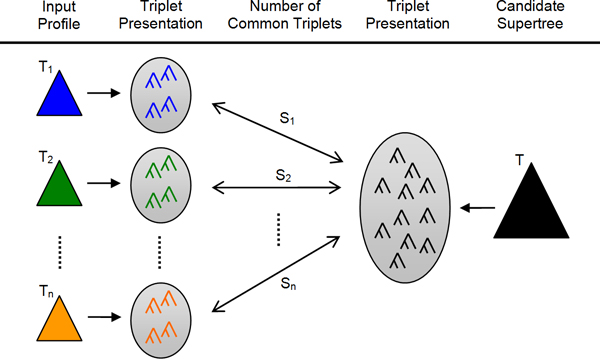
**Triplet supertree problem**. Given an input profile of *n *species trees (*T*_1_,..., *T*_*n*_), the triplet supertree problem is to find a supertree that maximizes the triplet-similarity score. The score for a supertree is calculated by first decomposing trees into their corresponding triplet presentations, then counting the number of common triplets between the supertree and each input tree (*S*_1_,..., *S*_*n*_), and finally aggregating all the counts. The triplet-similarity score for the candidate supertree *T *with respect to the input profile is therefore $\sum_{i=1}^{n}S_{i}$.

The triplet supertree problem makes use of the fact that every rooted tree can be equivalently represented by a set of triplet trees [17]. A triplet tree is a rooted fully binary tree over three taxa. Thus, a *triplet-similarity *measure can be defined between two rooted trees that is the cardinality of the intersection of their triplet presentations. This measure can be extended to measure the similarity from a collection of rooted input trees to a rooted supertree, by summing up the triplet-similarities for each input tree and the supertree. The triplet supertree problem is to find a supertree that maximizes the triplet-similarity for a given collection of input trees. Figure 1 illustrates the triplet supertree problem.

#### Hill-climbing heuristics

We introduce hill-climbing heuristics to solve the triplet supertree problem. Hill-climbing heuristics have been successfully applied to several intrinsically complex supertree problems. In these heuristics a tree graph is defined for the given set of input trees and some, typically symmetric, tree-edit operation. The nodes in the tree graph are the phylogenetic trees over the overall taxon set of the input trees. An edge adjoins two nodes exactly if the corresponding trees can be transformed into each other by the tree edit operation. The cost of a node in the graph is the measurement from the input trees to the tree represented by the node under the particular supertree problems optimization measurement. For the triplet supertree problem, the cost of a node in the graph is the triplet-similarity from the input trees to the tree represented by the node. Given a starting node in the tree graph, the heuristic's task is to find a maximal-length path of steepest ascent in the cost of its nodes and to return the last node on such a path. This path is found by solving the local search problem for every node along the path. The local search problem is to find a node with the maximum cost in the neighborhood (all adjacent nodes) of a given node. The neighborhood searched depends on the edit operation. Edit operations of interest are SPR and TBR [17]. We defer the definition of these operations to the next section. The best known run times (naïve solutions) for the SPR and TBR based local search problems under the triplet-similarity measurement are *O*(*kn*^4^) and *O*(*kn*^5^) respectively, where *k *is the number of input gene trees and *n *is the number of taxa present in the input gene trees.

### Contribution of the manuscript

We introduce algorithms that solve the local SPR and TBR based search problems for our triplet supertree heuristics in times *O*(*n*^3^) and *O*(*n*^3^) respectively, with an initial pre-processing time of *O*(*kn*^3^). These algorithms allow true large-scale phylogenetic analyses using hill-climbing heuristics for the triplet supertree problem. Finally, we demonstrate the performance of our SPR and TBR based hill-climbing heuristics in comparative studies on two large published data sets.

## Methods

Initially, for each possible triplet over the set of all taxa we count and store the frequency displayed by all the input trees in *O*(*kn*^3^) time. Then, for each local search problem, we use dynamic programming to efficiently pre-process necessary triplet counts in *O*(*n*^3^) time. By exploiting the structural properties of SPR and TBR related to triplet-similarity, we are able to use these triplet counts to compute the differences in triplet-similarity for all SPR and TBR neighborhoods, each in *O*(*n*^3^) time.

### Basic definitions, notations, and preliminaries

In this section we introduce basic definitions and notations and then define preliminaries required for this work. For brevity the proofs of Lemmas 2–6 are omitted, but available on request.

#### Basic definitions and notations

A *tree T *is a connected graph with no cycles, consisting of a node set *V*(*T*) and an edge set *E*(*T*). *T *is *rooted *if it has exactly one distinguished node called the *root *which we denote by Ro(*T*).

Let *T *be a rooted tree. We define ≤_*T *_to be the partial order on *V*(*T*) where *x *≤_*T *_*y *if *y *is a node on the path between Ro(*T*) and *x*. If *x *≤_*T *_*y *we call *x *a *descendant *of *y*, and *y *an *ancestor *of *x*. We also define *x *<_*T *_*y *if *x *≤_*T *_*y *and *x *≠ *y*, in this case we call *x *a *proper descendant *of *y*, and *y *a *proper ancestor *of *x*.

The set of minima under ≤_*T *_is denoted by Le(*T*) and its elements are called *leaves*. If {*x*, *y*} ∈ *E*(*T*) and *x *≤_*T *_*y *then we call *y *the *parent *of *x *denoted by Pa_*T*_(*x*) and we call *x *a *child *of *y*. The set of all children of *y *is denoted by Ch_*T*_(*y*). If two nodes in *T *have the same parent, they are called *siblings*. The *least common ancestor *of a non-empty subset *L *⊆ *V*(*T*), denoted as *lca*_*T*_(*L*), is the unique smallest upper bound of *L *under ≤_*T*_.

If *e *∈ *E*(*T*), we define *T*/*e *to be the tree obtained from *T *by identifying the ends of *e *and then deleting *e*. *T*/*e *is said to be obtained from *T *by *contracting e*. If *v *is a vertex of *T *with degree one or two, and *e *is an edge incident with *v*, the tree *T*/*e *is said to be obtained from *T *by *suppressing v*.

The *restricted subtree *of *T *induced by a non-empty subset *L *⊆ *V*(*T*), denoted as *T*|*L*, is the tree induced by *L *where all internal nodes with degree two are suppressed, with the exception of the root node. The *subtree *of *T *rooted at node *y *∈ *V*(*T*), denoted as *T*_*y*_, is the restricted subtree induced by {*x *∈ *V*(*T*): *x *≤_*T *_*y*}.

*T *is fully *binary *if every node has either zero or two children. Throughout this paper, the term tree refers to a rooted fully binary tree.

#### The triplet supertree problem

We now introduce necessary definitions to state the triplet supertree problem. A *triplet *is a rooted binary tree with three leaves. A triplet *T *with leaves *a*, *b*, and *c *is denoted *ab*|*c *if *lca*_*T*_({*a*, *b*}) is a proper descendant of the root. Note that we do not distinguish between *ab*|*c *and *ba*|*c*. The set of all triplets of a tree *T*, denoted as Tr(*T*), is {*ab*|*c *: *T*|{*a*, *b*, *c*} = *ab*|*c*}. The set of *common triplets *between two trees *T*_1 _and *T*_2_, denoted as *S*(*T*_1_, *T*_2_), is Tr(*T*_1_) ∩ Tr(*T*_2_). A *profile P *is a tuple of trees (*T*_1_,..., *T*_*n*_), we extend the definition of leaf set to profiles as $\text{Le}(P)=\cup_{i=1}^{n}\text{Le}(T_{i})$. Let *P *be a profile, we call T* a *supertree *of *P *if Le(T*) = Le(*P*).

We are now ready to define the triplet supertree problem (Fig. 1).

**Definition 1 **(Triplet similarity). *Given a profile P *= (*T*_1_,..., *T*_*n*_) and a supertree T* *of P, we define the *triplet-similarity score $S(P,T^{*})=\sum_{i=1}^{n}\left|S(T_{i},T^{*})\right|$.

**Problem 1 **(The triplet supertree problem). Given a profile P, find a supertree T* *that maximizes S*(*P*, T*). We call any such T* *a *triplet supertree.

**Theorem 1 **([14]). *The triplet supertree problem is NP-hard*.

#### Local search problems

**Figure 2:**
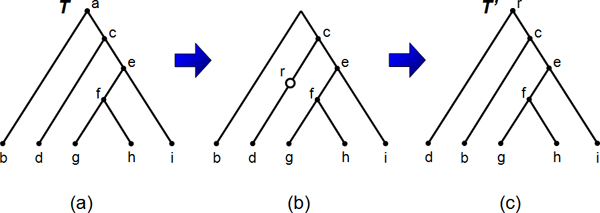
**Example of an RR operation**. Depicted is an example of an RR operation where *T' *= RR_*T*_(*d*). The original tree *T *is shown in (a). In (b), we first suppress the root node, and then introduce the new root node *r *above *d*. Finally we rearrange the tree so that *r *is at root, as in (c).

**Figure 3:**
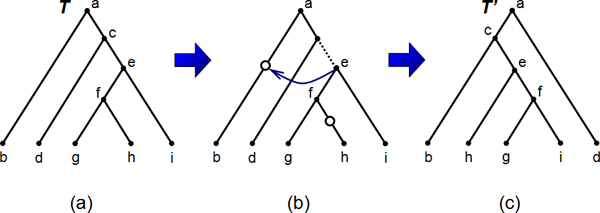
**Example of a TBR operation**. Depicted is an example of a TBR operation where *T' *= TBR_*T*_(*e*, *h*, *b*). The original tree *T *is shown in (a). In (b), we first remove the edge above *e*, that is we prune the subtree *T*_*e*_. Then we introduce a new node above *h *which will be the new root of the pruned subtree. We also introduce a new node above *b *– this is where we will reconnect the subtree back to *T*. Finally we rearrange the tree and obtain the resulting tree *T' *as in (c).

Here we first provide definitions for the re-root (RR), TBR, and SPR edit operations and then formulate the related local search problems. Figures 2 and 3 illustrate the RR and TBR edit operations respectively.

**Definition 2 **(RR operation). *Let T be a tree and x *∈ *V*(*T*). RR_*T*_(*x*) *is defined to be the tree T if x *= Ro(*T*). *Otherwise*, RR_*T*_(*x*) *is the tree that is obtained from T by (i) suppressing *Ro(*T*), *and (ii) subdividing the edge *{Pa_*T*_(*x*), *x*} *by a new root node. We define the following extension*:

RR_*T *_= ∪_*x*∈*V*(*T*)_{RR_*T*_(*x*)}.

*Let x *≤_*T *_*v, we also define a partial *RR *operation *RR_*T*_(*v*, *x*) *by replacing T*_*v *_*with *${\text{RR}}_{T_{v}}$(*x*).

**Definition 3 **(TBR operation). *For technical reasons we first define for a tree T the *planted tree Pl(*T*) *that is the tree obtained by adding an additional edge, called *root edge, {*r*, Ro(*T*)} *to E*(*T*).

*Let T be a tree, e *= (*u*, *v*) ∈ *E*(*T*), *and X, Y be the connected components that are obtained by removing edge e from T where v *∈ *X and u *∈ *Y. We define *TBR_*T*_(*v*, *x*, *y*) *for x *∈ *X and y *∈ *Y to be the tree that is obtained from *Pl(*T*) *by first removing edge e, then replacing the component X by *RR_*X*_(*x*), *and then adjoining a new edge f between x' *= Ro(RR_*X*_(*x*)) *and Y as follows:*

*1. Create a new node y' that subdivides the edge *(Pa_*T*_(*y*), *y*).

*2. Adjoin the edge f between nodes x' and y'*.

*3. Suppress the node u, and rename x' as v and y' as u*.

*4. Contract the root edge*.

*We say that the tree *TBR_*T*_(*v*, *x*, *y*) *is obtained from T by a *tree bisection and reconnection (TBR) *operation that *bisects *the tree T into the components X*, *Y and reconnects them above the nodes x*, *y*.

*We define the following extensions for the *TBR *operation:*

*1*. TBR_*T*_(*v*, *x*) = ∪_*y*∈*Y *_TBR_*T*_(*v*, *x*, *y*)

*2*. TBR_*T*_(*v*) = ∪_*x*∈*X *_TBR_*T*_(*v*, *x*)

*3*. TBR_*T *_= ∪_(*u*, *v*)∈*E*(*T*) _TBR_*T*_(*v*)

An SPR operation for a tree *T *can be briefly described through the following steps: (i) prune some subtree *S *from *T*, (ii) add a root edge to the remaining tree *T'*, (iii) regraft *S *into an edge of the remaining tree *T'*, (iv) contract the root edge. For our purposes we define the SPR operation as a special case of the TBR operation.

**Definition 4 **(SPR operation). *Let T be a tree, e *= (*u*, *v*) ∈ *E*(*T*), *and X, Y be the connected components that are obtained by removing edge e from T where v *∈ *X and u *∈ *Y. We define *SPR_*T*_(*v*, *y*) *for y *∈ *Y to be *TBR_*T*_(*v*, *v*, *y*). *We say that the tree *SPR_*T*_(*v*, *y*) *is obtained from T by a *subtree prune and regraft (SPR) *operation that *prunes *subtree T*_*v *_*and *regrafts *it above node y*.

*We define the following extensions of the *SPR *operation:*

*1*. SPR_*T*_(*v*) = ∪_*y*∈*Y *_SPR_*T*_(*v*, *y*)

*2*. SPR_*T *_= ∪_(*u*, *v*)∈*E*(*T*) _SPR_*T*_(*v*)

**Problem 2 **(TBR Scoring (TBR-S)). Given a profile P and a supertree T of P, find a tree T* ∈ TBR_*T *_*such that *$S(P,T^{*})=max_{T^{\prime }\in {\text{TBR}}_{T}}S(P,T^{\prime })$

**Problem 3 **(TBR-Restricted Scoring (TBR-RS)). *Given a profile P, a supertree T of P, and *(*u*, *v*) ∈ *E*(*T*), find a tree T* ∈ TBR_*T*_(*v*) *such that *$S(P,T^{*})=max_{T^{\prime }\in {\text{TBR}}_{T}}S(P,T^{\prime })$

The problems SPR Scoring (SPR-S) and SPR-Restricted Scoring (SPR-RS) are defined analogously to the problems TBR-S and TBR-RS respectively.

Further, we observe that to solve any of these four local search problems, it is sufficient to find a tree within the neighborhood that gives the maximum increase on S(*P*, *T*), without calculating the value of each S(*P*, *T*) itself. With this observation, it is useful to give the following definition.

**Definition 5**. *Let P be a profile, T*_1 _*and T*_2 _*be two supertrees of P, we define the *score difference function, *denoted as *Δ_*P*_(*T*_1_, *T*_2_), *to be *S(*P*, *T*_2_) - S(*P*, *T*_1_).

### Solving the SPR-RS and SPR-S problems

We first show how to solve the SPR-RS problem. Extending on this solution we introduce a new algorithm for the SPR-S problem.

#### Solving the *SPR*-RS problem

Given a profile *P*, a supertree *T *of *P*, and (*u*, *v*) ∈ *E*(*T*), we compute Δ_*P*_(*T*, *T'*) for each *T' *∈ SPR_*T*_(*v*) by first pruning and regrafting *T*_*v *_to Ro(*T*) and compute the score differences for each "move-down" operation, then traverse *T *in pre-order to obtain the tree that gives the maximum score difference. We first give a definition that helps us describe a single "move-down".

**Definition 6 **(Immediate Triplet). *Let T be a tree and v *∈ *V*(*T*), *an *immediate triplet *induced by v, denoted as yz *▷◁ *v*, *is a triplet yz*|*v where there exists nodes a, b *∈ *V*(*T*) *such that *Pa_*T*_(*y*) = Pa_*T*_(*z*) = *b and *Pa_*T*_(*b*) = Pa_*T*_(*v*) = *a*.

**Algorithm 1 **Algorithm for the SPR-RS problem

1: **procedure **SPR-RS(*P*, *T*, (*u*, *v*))

Input: A profile *P *= (*T*_1_,..., *T*_*n*_), a supertree *T *of *P*, and (*u*, *v*) ∈ *E*(*T*)

Output: T* ∈ SPR_*T*_(*v*), and Δ_*P*_(*T*, T*)

2:   *r *← Ro(*T*)

3:   $\hat{T}$ ← SPR_*T*_(*v*, *r*)

4:   Call MovedownAndCompute(*P*, $\hat{T}$, *v*)

5:   Traverse the tree *T*_*r *_in pre-order to compute Δ_*P*_($\hat{T}$, *T'*) for each *T' *∈ SPR_*T*_(*v*) using the values computed by MovedownAndCompute

6:   T* ← *T' *∈ SPR_*T*_(*v*) such that $\Delta_{P}(T,T^{\prime })=max_{T^{\prime \prime }\in {\text{SPR}}_{T}}\Delta_{P}(\hat{T},T^{\prime \prime })$

7:   *d *← Δ_*P*_($\hat{T}$, T*) - Δ_*P*_($\hat{T}$, *T*)

8:   **return **(T*, *d*)

9: **end procedure**

10: **procedure **MOVEDOWNANDCOMPUTE(*P*, *T*, *v*)

Input: A profile *P*, a tree *T*, and *v *∈ *V*(*T*)

11:   *yz *▷◁ *v *← The immediate triplet induced by *v *in *T*

12:   **for all ***t *∈ {*y*, *z*} **do**

13:      *T' *← SPR_*T*_(*v*, *t*)

14:      Compute and store Δ_*P*_(*T*, *T'*)

15:      Call MovedownAndCompute(*P*, *T'*, *v*)

16:   **end for**

17: **end procedure**

It can be easily seen that Algorithm 1 is correctly solving the SPR-RS problem.

#### Solving the *SPR*-S problem

**Algorithm 2 **Algorithm for the SPR-S problem

1: **procedure **SPR-S(*P*, *T*)

Input: A profile *P *= (*T*_1_,..., *T*_*n*_), a supertree *T *of *P*

Output: T* ∈ SPR_*T*_, and Δ_*P*_(*T*, T*)

2:   **for all **(*u*, *v*) ∈ *E*(*T*) **do**

3:      Store the value of SPR-RS(*P*, *T*, (*u*, *v*))

4:   **end for**

5:   (T*, *d*) ← the stored value of SPR-RS calls that has the maximum score increase by traversing the tree *T *in post-order

6:   **return **(T*, *d*)

7: **end procedure**

Algorithm 2 gives a trivial extension of Algorithm 1 to solve the SPR-S problem.

#### Computing Δ_*P*_(*T*, *T'*) efficiently

Algorithm 1 assumed the computation of Δ_*P*_(*T*, *T'*) for each move-down operation (Line 14). In this section we show how to compute each Δ_*P*_(*T*, *T'*) efficiently by exploiting structural properties related to the triplet-similarity. We begin with some useful definitions.

**Definition 7**. *Let A, B, C be pairwise mutual exclusive leaf sets, we extend the triplet notation by AB*|*C *= {*ab*|*c *: *a *∈ *A*, *b *∈ *B*, *c *∈ *C*}. *Further, let u, v, w be three nodes in a tree T having no ancestral relationships, define uv*|_*T*_*w *= Le(*T*_*u*_) Le(*T*_*v*_)| Le(*T*_*w*_)

**Definition 8**. *The *Boolean value *of a statement ϕ, denoted as *⟦*ϕ*⟧, *is 1 if ϕ is true, 0 otherwise*.

**Definition 9**. *Given a profile P *= (*T*_1_,..., *T*_*n*_) *and distinct a, b, c *∈ Le(*P*), *we define the *triplet summation function *by*

$$\sigma_{P}(ab|c)=\sum_{i=1}^{n}〚ab|c\in \text{Tr}(T_{i})〛$$

*Let A, B, C *⊆ Le(*P*) *be pairwise mutual exclusive leaf sets, we extend the triplet summation function by*

$$\sigma_{P}(AB|C)=\sum_{a\in A,b\in B,c\in C}\sigma_{P}(ab|c)$$

*Further*, *let u*, *v*, *w be three nodes in a tree T having no ancestral relationships*, *we define*

*σ*_*P*, *T*_(*uv*|*w*) = *σ*_*P*_(*uv*|_*T*_*w*)

**Lemma 1**. *Let T be a tree and yz *▷◁ *v be an immediate triplet induced by a node v *∈ *V*(*T*). *If T' *= SPR_*T*_(*v*, *y*), *then*

(1) Tr(*T'*) = (Tr(*T*)\*yz*|_*T*_*v*) ∪ *vy*|_*T*_*z*

(1) *Proof*. Let *a*, *b*, *c *∈ Le(*T*), we consider the following cases:

1. If any one of *a*, *b*, *c *is not in the subtree $T_{{\text{Pa}}_{T}(v)}$, then *T'*|{*a*, *b*, *c*} = *T*|{*a*, *b*, *c*}. Since both *yz*|_*T*_*v *and *vy*|_*T*_*z *only contain triplets formed under the subtree $T_{{\text{Pa}}_{T}(v)}$, *T*|{*a*, *b*, *c*} and only this triplet resolution of {*a*, *b*, *c*} is in both sides of equality.

2. Consider three subtrees *T*_*v*_, *T*_*y*_, *T*_*z*_. If *a*, *b*, *c *are all in one of the subtree, then since the subtrees *T*_*v*_, *T*_*y*_, *T*_*z *_do not change by the SPR operation, *T'*|{*a*, *b*, *c*} = *T*|{*a*, *b*, *c*}. Since both *yz*|_*T*_*v *and *vy*|_*T*_*z *only contain triplets that are formed by one leaf from each of *T*_*v*_, *T*_*y*_, and *T*_*z *_subtrees, *T*|{*a*, *b*, *c*} and only this triplet resolution of {*a*, *b*, *c*} is in both sides of equality.

3. Consider three subtrees *T*_*v*_, *T*_*y*_, *T*_*z*_. If two leaves of {*a*, *b*, *c*} are in one subtree and the other leaf is in another subtree, and suppose WLOG that {*a*, *b*} are in one subtree, then we observe that *lca*_*T*_({*a*, *b*}) <_*T *_*lca*_*T*_({*a*, *b*, *c*}) and *lca*_*T'*_({*a*, *b*}) <_*T' *_*lca*_*T'*_({*a*, *b*, *c*}), so *T'*|{*a*, *b*, *c*} = *T*|{*a*, *b*, *c*} = *ab*|*c*.

Also, as in Case 2, both *yz*|_*T*_*v *and *vy*|_*T*_*z *does not contain triplet formed by {*a*, *b*, *c*}, so *T*|{*a*, *b*, *c*} and only this triplet resolution of {*a*, *b*, *c*} is in both sides of equality.

4. If each of *T*_*v*_, *T*_*y*_, and *T*_*z *_contains exactly one leaf in {*a*, *b*, *c*}, and suppose WLOG that *a *∈ Le(*T*_*v*_), *b *∈ Le(*T*_*y*_), and *c *∈ Le(*T*_*z*_). Then *T*|{*a*, *b*, *c*} = *bc*|*a *and *T'*|{*a*, *b*, *c*} = *ab*|*c*. Also we observe that *bc*|*a *∈ *yz*|_*T*_*v *and *ab*|*c *∈ *vy*|_*T*_*z*, therefore RHS, and hence both sides contain *ab*|*c *and only this resolution of {*a*, *b*, *c*}.

**Lemma 2**. *Given a profile P and a supertree T of P, let yz *▷◁ *v be an immediate triplet induced by a node v *∈ *V*(*T*). *If T' *= SPR_*T*_(*v*, *y*), *then*

(2) Δ_*P *_(*T*, *T'*) = *σ*_*P*, *T*_(*vy*|*z*) - *σ*_*P*, *T*_(*yz*|*v*)

**Lemma 3**. *Given a profile P and a supertree T of P, let distinct v, b *∈ *V*(*T*) *such that v *≰_*T *_*b*, *b *≰_*T *_*v*, *and *Ch_*T*_(*b*) = {*y*, *z*}. *If T*_1 _= SPR_*T*_(*v*, *b*) *and T*_2 _= SPR_*T*_(*v*, *y*), *then*

(3) Δ_*P*_(*T*_1_, *T*_2_) = *σ*_*P*, *T*_(*vy*|*z*) - *σ*_*P*, *T*_(*yz*|*v*)

**Lemma 4**. *Given a profile P and a supertree T of P, let v, b *∈ *V*(*T*) *such that *Pa_*T *_(Pa_*T*_(*v*)) ≤_*T *_*b*, *and let *∏ = {*x *∈ *V*(*T*): *v *<_*T *_*x *<_*T *_*b*}. *Define C *= ∪_*x*∈∏ _{c∈Ch_*T*_(*x*):v≰_T_c}. *Let *Ch_*T*_(*b*) = {*y*, *z*}, *and WLOG suppose y *∉ ∏. *If T*_1 _= SPR_*T*_(*v*, *b*) *and T*_2 _= SPR_*T*_(*v*, *y*), *then*

(4) $$\Delta_{P}(T_{1},T_{2})=\sum_{x\in C}\left[\sigma_{P,T}(vy|x)-\sigma_{P,T}(yx|v)\right]$$

Equations (3) and (4) provide computations for all SPR-RS neighborhoods (Line 14 of Algorithm 1). We now show how to compute them efficiently.

**Algorithm 3 **Algorithm to compute triplet summation function

1: **procedure **PREPROCESSTRIPLETSUM(*P*)

Input: A profile *P *= (*T*_1_,..., *T*_*n*_)

2:   Initialize all values of *σ*_*P *_to 0

3:   **for ***i *= 1 to *n ***do**

4:      **for all ***u *∈ *V*(*T*_*i*_) in post-order, *u *∉ Le(*T*_*i*_) **do**

5:         {*v*, *w*} ← Ch_*T*_(*u*)

6:         **for all **{*x*, *y*} ∈ Le(*T*_*v*_), *z *∈ Le(*T*_*w*_) **do**

7:            Increment *σ*_*P*_(*xy*|*z*)

8:         **end for**

9:         **for all **{*x*, *y*} ∈ Le(*T*_*w*_), *z *∈ Le(*T*_*v*_) **do**

10:            Increment *σ*_*P*_(*xy*|*z*)

11:         **end for**

12:      **end for**

13:   **end for**

14: **end procedure**

Given a profile *P *= (*T*_1_,..., *T*_*n*_), we start by computing the triplet summation function *σ*_*P *_for all triplets, as shown by Algorithm 3.

**Algorithm 4 **Algorithm to compute extended triplet summation function

1: **procedure **PREPROCESSEXTENDEDTRIPLETSUM(*P*, *T*)

Input: A profile *P *= (*T*_1_,..., *T*_*n*_), a supertree *T *of *P*

2:   **for all ***u *∈ *V*(*T*) in post-order **do**

3:      **for all ***v *∈ *V*(*T*) in post-order after *u*, *lca*_*T*_({*u*, *v*}) ∉ {*u*, *v*} **do**

4:         **for all ***w *∈ *V*(*T*) in post-order after *v*, *lca*_*T*_({*u*, *v*, *w*}) ∉ {*u*, *v*, *w*} **do**

5:            **if ***w *∈ Le(*T*) **then**

6:               **if ***v *∈ Le(*T*) **then**

7:                  **if ***u *∈ Le(*T*) **then**

8:                     *σ*_*P*, *T*_(*uv*|*w*) ← *σ*_*P*_(*uv*|*w*)

9:                     *σ*_*P*, *T*_(*uw*|*v*) ← *σ*_*P*_(*uw*|*v*)

10:                     *σ*_*P*, *T*_(*vw*|*u*) ← *σ*_*P*_(*vw*|*u*)

11:                  **else**

12:                     {*u*_1_, *u*_2_} ← Ch_*T*_(*u*)

13:                     *σ*_*P*, *T*_(*uv*|*w*) ← *σ*_*P*, *T*_(*u*_1_*v*|*w*) + *σ*_*P*, *T*_(*u*_2_*v*|*w*)

14:                     *σ*_*P*, *T*_(*uw*|*v*) ← *σ*_*P*, *T*_(*u*_1_*w*|*v*) + *σ*_*P*, *T*_(*u*_2_*w*|*v*)

15:                     *σ*_*P*, *T*_(*vw*|*u*) ← *σ*_*P*, *T*_(*vw*|*u*_1_) + *σ*_*P*, *T*_(*vw*|*u*_2_)

16:                  **end if**

17:               **else**

18:                     {*v*_1_, *v*_2_} ← Ch_*T*_(*v*)

19:                     *σ*_*P*, *T*_(*uv*|*w*) ← *σ*_*P*, *T*_(*uv*_1_|*w*) + *σ*_*P*, *T*_(*uv*_2_|*w*)

20:                     *σ*_*P*, *T*_(*uw*|*v*) ← *σ*_*P*, *T*_(*uw*|*v*_1_) + *σ*_*P*, *T*_(*uw*|*v*_2_)

21:                     *σ*_*P*, *T*_(*vw*|*u*) ← *σ*_*P*, *T*_(*v*_1_*w*|*u*) + *σ*_*P*, *T*_(*v*_2_*w*|*u*)

22:                  **end if**

23:                  **else**

24:                     {*w*_1_, *w*_2_} ← Ch_*T*_(*w*)

25:                     *σ*_*P*, *T*_(*uv*|*w*) ← *σ*_*P*, *T*_(*uv*|*w*_1_) + *σ*_*P*, *T*_(*uv*|*w*_2_)

26:                     *σ*_*P*, *T*_(*uw*|*v*) ← *σ*_*P*, *T*_(*uw*_1_*|v*) + *σ*_*P*, *T*_(*uw*_2_*|v*)

27:                     *σ*_*P*, *T*_(*vw*|*u*) ← *σ*_*P*, *T*_(*vw*_1_|*u*) + *σ*_*P*, *T*_(*vw*_2_|*u*)

28:                  **end if**

29:               **end for**

30:            **end for**

31:         **end for**

32: **end procedure**

Next, we compute the extended triplet summation function *σ*_*P*, *T*_, for all nodes *u*, *v*, *w *in *T *having no ancestral relationships, as shown by Algorithm 4.

**Algorithm 5 **Algorithm to compute score difference function

1: **procedure **PREPROCESSSCOREDIFFERENCE(*P*, *T*)

Input: A profile *P *= (*T*_1_,..., *T*_*n*_), a supertree *T *of *P*

2:   **for all ***v *∈ *V*(*T*) **do**

3:      **for all **(*b*, *y*) ∈ *E*(*T*): *b *≰_*T *_Pa_*T*_(*v*) **do**

4:         Let Ch_*T*_(*b*) = {*y*, *z*}

5:         Let *T*_1 _= SPR_*T*_(*v*, *b*), *T*_2 _= SPR_*T*_(*v*, *y*)

6:         **if ***v *≤_*T *_*b ***then**

7:            **for all ***p *∈ *V*(*T*): *v *<_*T *_*p *<_*T *_*b ***do**

8:               Let *x *∈ Ch_*T *_(*p*) where *v *≰_*T *_*x*

9:               Δ_*P*_(*T*_1_, *T*_2_) ← Δ_*P*_(*T*_1_, *T*_2_) + *σ*_*P*, *T*_(*vy*|*x*) - *σ*_*P*, *T*_(*yx*|*v*)

10:            **end for**

11:         **else**

12:            *Δ*_*P*_(*T*_1_, *T*_2_) ← *σ*_*P*, *T*_(*vy*|*z*) - *σ*_*P*, *T*_(*yz*|*v*)

13:         **end if**

14:      **end for**

15:   **end for**

16: **end procedure**

Finally, we compute the score difference function Δ_*P*_(*T*_1_, *T*_2_) for all *v *∈ *V*(*T*) and (*b*, *y*) ∈ *E*(*T*) where *b *≰_*T *_Pa_*T*_(*v*) such that *T*_1 _= SPR_*T*_(*v*, *b*) and *T*_2 _= SPR_*T*_(*v*, *y*). This is shown by Algorithm 5.

#### Time complexity

We describe the time complexity for our solution for the SPR-S problem. First, we run Algorithm 3 once for the entire heuristic run, which takes *O*(*kn*^3^) where *k *is the number of input trees and *n *is the number of taxa present in the input trees. Then, for each SPR-S problem, we begin by pre-processing necessary counts using Algorithm 4 and 5, each takes time *O*(*n*^3^). These pre-processed computations allow Algorithm 1 to run in *O*(*n*) time. Finally, Algorithm 2 issues *O*(*n*) calls to Algorithm 1, so overall it takes *O*(*n*^2^) time. Including the pre-processing steps, we see that solving the SPR-S problem takes *O*(*n*^3^) time, with an expense of *O*(*kn*^3^) at the beginning of the entire heuristic run.

### Solving the TBR-RS and TBR-S problems

We extend our solutions of SPR-RS and SPR-S problems to solve TBR-RS and TBR-S problems.

#### Solving the *TBR*-RS problem

We observe that a TBR operation can be viewed as an SPR operation followed by an RR operation. We exploit this structural property further and establish some lemmas which helps us compute the score differences for all TBR operations in the TBR-RS neighborhood.

**Lemma 5**. *Given a profile P, a supertree T of P, and a valid *TBR *operation on T where T' *= TBR_*T*_(*v*, *x*, *y*), *then*

(5) Δ_*P*_(*T*, *T'*) = Δ_*P*_(*T*, SPR_*T*_(*v*, *y*)) + Δ_*P*_(*T*, RR_*T*_(*v*, *x*))

Lemma 5 implies that given a subtree, we can find the best TBR-RS neighborhood by finding the best SPR-RS neighborhood and apply the best re-rooting for the subtree regardless of which SPR operation was chosen. Further, we note that RR is a special case of SPR operation by the following lemma.

**Lemma 6**. *Given a profile P and a supertree T of P, let xy *▷◁ *z be an immediate triplet induced by a node z *∈ *V*(*T*) *and *Pa_*T*_(*z*) = *v*, *then*

(6) RR_*T*_(*v*, *x*) = SPR_*T*_(*z*, *y*)

This means that we can reuse Lemmas 2, 3, 4 and their corresponding algorithms to compute the score differences of all move-downs in terms of re-rooting. Hence, given a subtree the algorithm would first compute the best re-rooting and its score difference, then simply join this RR operation with the best SPR operation in the SPR-RS neighborhood.

#### Solving the *TBR*-S problem

Similar to solving the SPR-S problem, we can solve the TBR-S problem given the solution to the TBR-RS problem in the previous section.

#### Time complexity

We perform the same steps to solve the SPR-S problem, and additionally by utilizing Lemmas 5 and 6 we compute and store the best re-rooting for each subtree, which takes *O*(*n*^3^) time. Overall, solving the TBR-S problem still takes *O*(*n*^3^) time.

## Results and discussion

We examined the performance of our new triplet heuristics by comparing with two other supertree methods, MRP and modified Min-Cut (MMC; [20]), using published data sets from marsupials [21] and Cetartiodactyla [22]. MRP is the most widely applied supertree method [3]. However, MRP supertrees, like triplet supertrees, are intrinsically hard to compute. Therefore they are estimated using heuristics, which do not guarantee an optimal solution. In contrast, MMC supertrees can be computed exactly in polynomial time, and therefore, it has been suggested that MMC will be useful for building very large phylogenies [20]. We evaluated each of the supertree methods using the triplet-similarity and the maximum agreement subtree (MAST) similarity [23] between the input trees and the supertrees. Furthermore, we measured the parsimony score of each computed supertree based on its binary matrix representation. For the marsupial data set, we also compared our results to published results using the max cut (MXC) supertree algorithm [24]. MXC is a modification of MMC that provides a heuristic approach based on the triplet supertree problem. There is currently no publicly available implementation of MXC (S. Snir, pers. comm.), and therefore, we were unable to apply it to the other data set.

Triplet supertrees were constructed using the programs TH(SPR) and TH(TBR) that implement hill-climbing heuristics based on our efficient local search algorithms for SPR and TBR branch swapping respectively. MRP supertrees were constructed using the parsimony hill-climbing heuristic implemented in PAUP* [9] with TBR branch swapping. We found very little difference in the results of MRP analyses when we collapsed zero-length branches and when we forced all MRP trees to be binary. We report the results from analyses that collapsed zero-length branches. All hill-climbing analyses were executed on 20 initial random addition sequence replicate trees and saving a single best tree per replicate. MMC supertrees were constructed using a program [20] supplied by Rod Page.

Our new triplet heuristics seek the supertrees with the maximum number of identical triplets to the collection of input trees, and indeed, we find they both outperform all other methods based on triplet-similarity in all data sets (Table 1). Furthermore, all the triplet supertree analyses were completed within 15 minutes using a Kensington quad-core 2.66 GHz Linux-based machine, demonstrating that our heuristics make the triplet supertree problem extremely tractable for large-scale analyses. Both triplet heuristics and the MRP heuristic perform much better than the exact MMC algorithm, based on triplet-similarity and MAST-similarity. The MXC algorithm suffers from the lack of an available implementation; however, in our single comparison with the published results, this algorithm does not perform as well as either the MRP heuristic or our triplet heuristics.

**Table 1:** Results of supertree analyses of two empirical data sets. Note that a bolded number represents the best found score for each measurement in a data set.

**Data Set**	**Method**	**Triplet-similarity**	**MAST-similarity**	**Pars. Score**
**Marsupial **[21] 158 input trees 267 taxa	MMC	51.73 %	54.20 %	3901
	
	MXC	≈ 96 %	≈ 66 %	N/A
	
	MRP	98.29 %	**71.70 **%	**2274**
	
	TH(SPR)	**98.99 **%	70.50 %	2317
	
	TH(TBR)	**98.99 **%	70.70 %	2317

**Cetartiodactyla **[22] 201 input trees 290 taxa	MMC	70.03 %	54.20 %	4929
	
	MRP	95.84 %	**65.40 **%	**2603**
	
	TH(SPR)	**97.28 **%	63.40 %	2754
	
	TH(TBR)	**97.28 **%	63.50 %	2754

Although the difference in the triplet-similarity score between the triplet heuristics and the MRP heuristic is always less than 2% (Table 1), due to the extremely large-number of triplets, even these apparently small differences represent large differences in tree topologies. For example, in the marsupial data set, the 0.7% difference in triplet-similarity represents over 17,400 triplets. The comparison of the triplet heuristics and the MRP heuristic also demonstrates that optimizing the parsimony score of the matrix representation of input trees is not directly correlated with optimizing the triplet-similarity of input trees and the supertree; supertrees with smaller (better) parsimony scores have lower (worse) triplet-similarity (Table 1).

Our experiments also demonstrate that the supertree with the best triplet-similarity is not necessarily best in terms of MAST-similarity. In fact, MRP outperforms the triplet heuristics in terms of the MAST-similarity to the input trees (Table 1). It is not intuitive that a parsimony analysis on a matrix representation of the input trees (MRP) is a valid or useful approach to infer the most accurate supertrees (but see [25]). The popularity of MRP is probably based more on the availability of programs that implement fast heuristics rather than a belief that parsimony on a matrix representation of input trees is the ideal supertree optimality criterion. However, MRP performs well in simple simulation experiments [26,27] and in analyses of empirical data (e.g., [13]), and it clearly can be an effective supertree method. Since it is not obvious whether it is better to find supertrees that maximize accuracy in terms of triplet-similarity, MAST-similarity, or some other tree similarity measure like the Robinson-Foulds distance, we suggest that the triplet heuristics are an informative complement to the MRP method. Both approaches can provide supertrees that represent different, and equally valid, perspectives on accuracy.

## Conclusion

Despite the inherent complexity of the triplet supertree problem, we have shown that it can be addressed effectively by using hill-climbing heuristics. We introduced efficient algorithms for standard local search problems that are solved by these heuristics. Our algorithms greatly improve on the best known (naïve) solutions for these search problems. This in turn makes hill-climbing heuristics for the triplet supertree problem applicable for large-scale phylogenetic studies.

We demonstrate the performance of an implementation of our hill-climbing heuristics. In analyses of two empirical data sets, our triplet heuristics quickly found supertrees that contained more triplets in common with the input trees than supertrees found by MRP, MMC, or MXC. These results demonstrate not only that our heuristics for the triplet supertree problem make it a valuable alternative to standard supertree methods. They also demonstrate that developing new supertree heuristics that directly seek to optimize the accuracy of the supertree with respect to the input trees can enhance our ability to infer with accuracy large sections of the tree of life.

The algorithmic ideas developed in this work might set base for theoretical properties that identifies a much broader class of local search objectives, which can be solved more efficiently. This could lead to other powerful supertree heuristics. However, it remains an open problem if our solutions for the SPR and TBR based local search problems for the triplet supertree problem can be improved further.

## Competing interests

The authors declare that they have no competing interests.

## Authors' contributions

HTL designed the triplet heuristics, implemented programs TH(SPR) and TH(TBR), and carried out the experiments. JGB led the analysis of the experimental results. OE inspired the triplet heuristics and supervised the project. All authors contributed to the writing of this manuscript, and have read and approved the final manuscript.
